# Supplementary material for: Effects of 12 Weeks Cosmos caudatus Supplement among Older Adults with Mild Cognitive Impairment: A Randomized, Double-Blind and Placebo-Controlled Trial
Source: Nutrients. 2021 Jan 29;13(2):434. doi: 10.3390/nu13020434 (PMC7912368; doi:10.3390/nu13020434)
Supplement: Supplementary file 1 [file nutrients-13-00434-s001.pdf]

Supplementary 1

Table 1. Baseline blood biochemical profiles between CC supplement group and placebo group subjects [presented as mean ± standard deviation]

Parameter	CC supplement (n=24)	Placebo (n=24)	Total (n=48)	Normal range	p-value
Fasting blood sugar (mmol/L)	5.77 ± 2.05	5.67 ± 1.63	5.72 ± 1.83	3.9-5.6	0.856
Urea (mmol/L)	5.15 ± 1.36	4.62 ± 1.08	4.88 ± 1.24	1.7-8.4	0.144
Creatinine (µmol/L)	69.30 ± 14.91	66.17 ± 18.32	67.70 ± 16.63	62-115	0.524
Calcium (mmol/L)	2.28 ± 0.10	2.27 ± 0.08	2.27 ± 0.09	2.12-2.52	0.742
Inorganic phosphate (mmol/L)	1.21 ± 0.13	1.25 ± 0.14	1.23 ± 0.13	0.78-1.65	0.295
Uric acid (mmol/L)	0.32 ± 0.07	0.36 ± 0.09	0.34 ± 0.08	0.20-0.42	0.112
Sodium (mmol/L)	139.61 ± 4.44	139.83 ± 2.35	139.72 ± 3.49	137-150	0.828
Potassium (mmol/L)	4.80 ± 0.47	4.66 ± 0.28	4.73 ± 0.39	3.5-5.3	0.234
Chloride (mmol/L)	102.04 ± 4.51	103.13 v 2.82	102.60 ± 3.75	96-108	0.328
Total cholesterol (mmol/L)	5.42 ± 1.01	5.25 ± 1.03	5.33 ± 1.01	<5.2	0.566
HDL(mmol/L)	1.52 ± 0.35	1.61 ± 0.37	1.57 ± 0.36	>1.04	0.387
LDL (mmol/L)	3.30 ± 0.84	2.98 ± 0.96	3.13 ± 0.91	<2.6	0.230
Triglyceride (mmol/L)	1.34 ± 0.79	1.45 ± 0.62	1.40 ± 0.71	<1.7	0.624
Total cholesterol to HDL ratio	3.68 ± 0.78	3.36 ± 0.81	3.51 ± 0.80	<5.0	0.173
Total protein (g/L)	70.91 ± 7.84	69.17 ± 2.82	70.02 ± 5.85	57-82	0.311
Albumin (g/L)	42.96 ± 2.88	43.29 ± 2.40	43.13 ± 2.63	32-48	0.667
Globulin (g/L)	28.00 ± 8.03	25.88 ± 3.57	26.91 ± 6.20	20-50	0.244
Albumin to globulin ratio	1.61 ± 0.33	1.71 ± 0.28	1.66 ± 0.31	1.2-2.5	0.273
Total bilirubin (µmol/L)	12.61 ± 5.81	11.83 ± 4.27	12.21 ± 5.04	3-19	0.603
AST (IU/L)	27.13 ± 12.22	23.21 ± 5.43	25.13 ± 9.49	0-40	0.159
ALP (IU/L)	75.91 ± 22.00	70.58 ± 13.76	73.19 ± 18.26	39-117	0.323
ALT (IU/L)	25.35 ± 4.10	23.13 ± 4.89	24.21 ± 19.75	0-40	0.704
GGT (IU/L)	28.35 ± 8.6	24.00 ± 5.22	26.13 ± 2.62	<73	0.516

Not significant at p>0.05

HDL: high density lipoprotein; LDL: low-density lipoprotein; AST: aspartate aminotransferase; ALP: alkaline phosphatase; ALT: alanine transferase; GGT: gamma-glutamyl transferase
